# Mitochondrial DNA analysis in primary congenital glaucoma

**Published:** 2010-03-24

**Authors:** Mukesh Tanwar, Tanuj Dada, Ramanjit Sihota, Rima Dada

**Affiliations:** 1 Laboratory for Molecular Reproduction and Genetics, Department of Anatomy, All India Institute of Medical Sciences, Ansari Nagar, New Delhi, India; 2 Dr. R.P. Centre for Ophthalmic Sciences, All India Institute of Medical Sciences, Ansari Nagar, New Delhi, India

## Abstract

**Purpose:**

To screen mitochondrial DNA (mtDNA) for nucleotide variations in primary congenital glaucoma (PCG).

**Methods:**

The entire coding region of the mitochondrial genome was amplified by polymerase chain reaction from 35 PCG patients and 40 controls. The full mtDNA genome except the D-loop was sequenced. All sequences were analyzed against mitochondrial reference sequence NC_012920.

**Results:**

MtDNA sequencing revealed a total of 132 and 58 nucleotide variations in PCG and controls, respectively. Of 132 nucleotide variations, 42 (31.81%) were non-synonymous and 82 (62.12%) were synonymous changes, and 8 were in RNA genes. The highest number of nucleotide variations were recorded in complex I followed by complex IV, then complex V. Eight patients (22.85%) had potentially pathogenic mtDNA nucleotide changes and twenty (57.14%) had mtDNA sequence changes associated with elevated reactive oxygen species (ROS) production. Mitochondria not only constitute the energy-generating system in the cell, but are also critically involved in calcium signaling and apoptosis. Mitochondrial function can be affected by mutations in mitochondrial and nuclear DNA, chemical insults to components of the electron transport chain, and a lack of substrates such as oxygen. Mitochondrial dysfunction results in an excessive generation of free radicals and reduced mitochondrial respiration. Developing trabecular meshwork (TM) is deficient in antioxidant enzymes, and thus is more susceptible to oxidative stress (OS) induced damage. Previous studies have documented certain mtDNA sequence variations associated with elevated ROS levels and OS. Three such changes (G10398A, A12308G, and G13708A) were present in our patients. Elevated ROS may cause OS. This OS may further damage mtDNA and may cause decreased mitochondrial respiration. This may lead to impaired growth, development and differentiation of TM and consequently trabecular-dysgenesis, which is a characteristic feature of PCG. OS affects both TM and retinal ganglion cells (RGCs) and may be involved in the neuronal death affecting the optic nerve in glaucoma. There are several studies which point to mitochondrial dysfunction in different types of glaucoma and critically participate in RGC death. Recent studies also implicate mitochondrial dysfunction-associated OS as a risk factor for glaucoma patients. It has been reported that elevated hydrostatic pressure causes breakdown of the mitochondrial network by mitochondrial fission and induce cristae depletion and cellular ATP reduction in differentiated RGC-5 cells in vitro as well as in vivo.

**Conclusions:**

A total of 44 novel mtDNA variations were identified in this study. Non-synonymous mtDNA variations may adversely affect respiratory chain, impair OXPHOS pathway result in low ATP production, high ROS production and impair growth, development and differentiation of TM lead to trabecular-dysgenesis and consequently RGC’s death. Such cases with mtDNA variations and consequent OS may benefit by early diagnosis and prompt management by antioxidant therapy. This may delay OS induced injury to TM and RGCs and hence improve visual prognosis.

## Introduction

Glaucomas are a heterogeneous group of eye conditions with manifestation as early as birth to very late age of onset and are among most common cause of blindness worldwide, accounting for 15% of cases. Primary congenital glaucoma (PCG; OMIM 231300; provided in the public domain by the National Centre for Biotechnology Information, Bethesda, MD) is a severe form of glaucoma with manifestation at birth or early childhood. It is characterized by elevated intra-ocular pressure (IOP), and enlarged cornea and globe (buphthalmos) [1]. The only observable anatomic defect in PCG is trabecular-dysgenesis. This leads to impaired aqueous drainage, increased intraocular pressure, optic nerve damage, and may consequently lead to partial/permanent visual impairment. Progressive degeneration of retinal ganglion cells (RGCs) and their axons is the primary cause of glaucomatous visual loss. However, many aspects of this blinding disorder are still unclear and current treatment options are not sufficient to block neurodegenerative injury in these patients.

PCG is bilateral in 80% cases. The majority of PCG cases present within the first year of life out of which 25% are diagnosed in the neonatal period and in about 60% within first six months of life. The majority of PCG cases are sporadic. PCG is the most common type of pediatric glaucoma and accounts for 55% of pediatric glaucomas. The prevalence of PCG varies across ethnic communities ranging from 1 in 10,000–20,000 in the western populations [2] to 1 in 2,500 and 1 in 1,250 in the Saudi Arabian population [3] and Gypsy population of Slovakia [2], and 1 in 3,300 in Andhra Pradesh, India [4]. Early and reliable diagnosis of this disease is vital, so that appropriate and prompt treatment is initiated. This can improve the visual outcome and prevent visual loss.

Three genetic loci: GLC3A at 2p21, GLC3B at 1p36, and GLC3C at 14q24.3-q31.1 have been mapped for PCG [3,5,6]. Mutations in *CYP1B1* (GLC3A locus) have been found in PCG patients from different populations [3,7-10] It is estimated that all known loci/genes of glaucoma account for the minority of total cases of glaucoma, and thus, many other genes remain to be identified.

The role of mitochondrial DNA (mtDNA) mutations and oxidative stress (OS) has been reported in primary open angle glaucoma (POAG) [11,12]. Recent studies reported an increased frequency of mtDNA sequence changes in primary open angle glaucoma (POAG), primary angle closure glaucoma (PACG), and pseudoexfoliation glaucoma (PEG) compared to controls [11,13,14]. Therefore this study was planned with the aim to screen PCG cases for mitochondrial DNA variations.

## Methods

### Clinical examination and selection of cases

Primary congenital glaucoma cases (n=35) presenting at the Dr. R. P. Centre for Ophthalmic Sciences (AIIMS, New Delhi, India), were enrolled for this study, after ethical approval of the Institutional Review Board (IRB00006862; All India Institute of Medical Sciences, New Delhi, India). The diagnosis involved clinical ocular and systemic examination. Inclusion criteria of the patients were: increased corneal diameter (>12.0 mm), raised IOP (>21 mmHg) with presence/absence of Haab’s striae, and optic disc changes (where examination was possible). Symptoms of epiphora and photophobia were additional inclusion factors. The age of onset ranged from birth to 3 years. All patients with a history of blood transfusion, TORCH (Toxoplasmosis; Rubella; Cytomegalovirus; Herpes Simplex Virus) infection, and drug intake in the mother during pregnancy were excluded. Glaucoma cases other than PCG were also excluded. Detailed family history of ocular or other hereditary disorders up to three generations were taken, and pedigree charts were constructed. Forty ethnically matched normal individuals without any ocular disorders with IOP<20mmHg and corneal diameter <12×12mm were enrolled as controls.

### Sample collection and DNA isolation

Peripheral blood sample was collected from patients and controls by venipuncture after informed consent. Blood samples were collected in EDTA (EDTA) vaccutainers and stored in −80 °C (°C) until DNA isolation. DNA was isolated from whole blood using the phenol-chloroform method.

### Polymerase chain reaction (PCR) amplification and sequence analysis of the mitochondrial DNA coding region

The whole mitochondrial genome was amplified in all patients and controls using 24 pairs of primers [15]. PCR amplifications for all primer sets were performed in a 40 μl volume containing 1.0 μl of 20 μM stock solution for each primer, 100 ng of genomic DNA, 1 unit of Taq polymerase (Banglore Genei, Bengaluru, Karnataka, India), 0.1 mM of each dNTP, 4 μl of 10× PCR buffer (with 15 mM MgCl_2_), by means of 30 cycles of amplification, each consisting of 30 s denaturation at 94 °C, 30 s annealing at 56 °C and 1 min extension at 72 °C. Finally, and extension for 5 min at 72 °C was performed. Amplified PCR products were purified using a gel/PCR DNA fragments extraction kit (catalog number DF100; Geneaid Biotech Ltd., Sijhih City, Taiwan). Purified PCR products were sent for sequencing to MCLAB (Molecular Cloning Laboratories, South San Francisco, CA). The full mtDNA genome was sequenced except D-loop as D-loop is a hyper-variable region. All fragments were sequenced in both forward and reverse direction for confirmation. All sequence variants from both PCG patients and controls were compared to Human Mitochondrial reference sequence NC_012920 provided by the National Center for Biotechnology Information (NCBI) using ClustalW2 (multiple sequence alignment program for DNA; European Molecular Biology Laboratory (EMBL) – European Bioinformatics Institute (EBI).

### Prediction of pathogenecity

For prediction of pathogenic characteristics of all non-synonymous mtDNA changes two homology based programs PolyPhen (Polymorphism Phenotyping) and SIFT (Sorting Intolerant From Tolerant) analysis tool were used. PolyPhen structurally analyzes an amino acid polymorphism and predicts whether that amino acid change is likely to be deleterious to protein function [16-18]. The prediction is based on the position-specific independent counts (PSIC) score derived from multiple sequence alignments of observations in case of functional domain of protein and Predicted hydrophobic and transmembrane (PHAT) matrix element difference in case of transmembrane region of protein. PolyPhen scores of >2.0 indicate the polymorphism is probably damaging to protein function. Scores of 1.5–2.0 are possibly damaging, and scores of <1.5 are likely benign. SIFT is a sequence homology-based tool that sorts intolerant from tolerant amino acid substitutions and predicts whether an amino acid substitution in a protein will have a phenotypic effect [19-22]. SIFT is based on the premise that protein evolution is correlated with protein function. Positions important for function should be conserved in an alignment of the protein family, whereas unimportant positions should appear diverse in an alignment. Positions with normalized probabilities less than 0.05 are predicted to be deleterious and, those greater than or equal to 0.05 are predicted to be tolerated.

### Statistical analysis

Pearson χ^2^/Fisher’s exact test was applied to make a comparison between two groups (cases versus controls). P-values less than 0.05 were considered as significant. All tests were done using SPSS software for windows (version 11.5; SPSS Inc., Chicago, IL).

## Results

**Figure 1:**
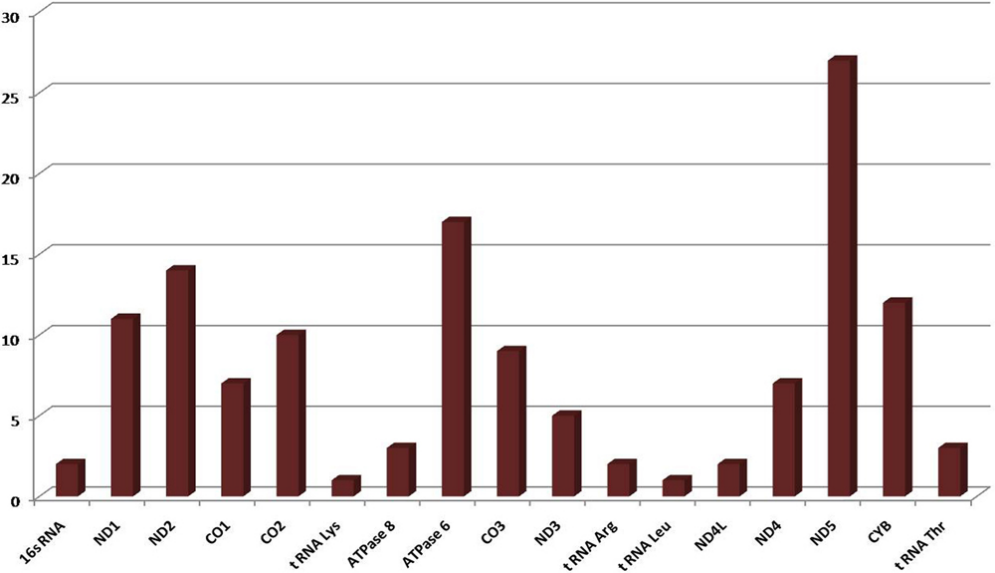
Bar diagram showing distribution of nucleotide variations in different mitochondrial genes in PCG. Abbrevations: ND1- NADH dehydrogenase subunit 1; ND2- NADH dehydrogenase subunit 2; ND3- NADH dehydrogenase subunit 3; ND4- NADH dehydrogenase subunit 4; ND4L- NADH dehydrogenase subunit 4L; ND5- NADH dehydrogenase subunit 5; CO1- cytochrome c oxidase I; CO2- cytochrome c oxidase II; CO3- cytochrome c oxidase III; ATPase6- ATP synthase subunit a (F-ATPase protein 6); ATPase8- ATP synthase protein 8; CYB- cytochrome B; tRNA- transfer ribo nucleic acid; rRNA- ribosomal ribonucleic acid.

MtDNA sequencing following whole genome amplification of mitochondrial DNA revealed a total of 132 nucleotide variations (Table 1) in PCG patients and 58 in controls (Table 2). Of the 132 nucleotide variations, 42 (31.81%) were non-synonymous, 82 (62.18%) were synonymous changes, and 8 were in RNA genes. In total, 23.48% (31/132) variations were novel out of which 41.93% (13/31) were non-synonymous (Table 1). A total of 66/132 (50.00%) variations were observed in complex I, 12/132 (9.09%) in complex III, 26/132 (19.69%) in complex IV, and 20/132 (15.15%) were in complex V (Figure 1). Out of the total variations reported, complex I had 31.81% (21/66) non-synonymous base changes, complex III had 25.00% (3/12), complex IV had 23.07% (6/26), and complex V had 55.00% (11/20) non-synonymous base changes. Of 58 variations in the controls, 14 were non-synonymous changes.

**Table 1:** Mitochondrial DNA variations observed in primary congenital glaucoma patients.

**Sample number**	**Genomic Position**	**Base Change**	**Gene /Location**	**Amino acid position**	**Codon change**	**Amino acid change**	**Change in protein**	**Syn/ Non-syn**	**Total number of patients with nt changes**	**GenBank accession number if novel**
1	2707	G>A	*16s RNA*	-	-	-	-	-	1	-
2	2790	Ins T	*16s RNA*	-	-	-	-	-	2	GU186097
3	3311	C>T	*ND1*	2	CCC>CTC	Pro>Leu	p.P2L	non-syn	1	GU186068
4	3316	G>A	*ND1*	4	GCC>ACC	Ala>Thr	p.A4T	non-syn	1	-
5	3335	T>C	*ND1*	10	ATT>ACT	IsoLeu>Thr	p.I10T	non-syn	1	-
6	3398	T>C	*ND1*	31	ATA>ACA	Met>Thr	p.M31T	non-syn	2	GU186069
7	3480	A>G	*ND1*	58	AAA>AAG	Lys>Lys	p.K58K	syn	2	-
8	3645	T>C	*ND1*	113	GTT>GTC	Val>Val	p.V113V	syn	1	-
9	3741	C>T	*ND1*	145	ACC>ACT	Thr>Thr	p.T145T	syn	1	-
10	3826	T>C	*ND1*	174	TTA>CTA	Leu>Leu	p.L174L	syn	1	-
11	3921	C>T	*ND1*	205	TCC>TCT	Ser>Ser	p.S205S	syn	2	-
12	4216	T>C	*ND1*	304	TAT>CAT	Tyr>His	p.Y304H	non-syn	1	-
13	4225	A>G	*ND1*	307	ATA>GTA	Met>Val	p.M307V	non-syn	1	-
14	4502	T>C	*ND2*	11	TCT>TCC	Ser>Ser	p.S11S	syn	1	GU186070
15	4561	T>C	*ND2*	31	GTA>GCA	Val>Ala	p.V31A	non-syn	1	-
16	4580	G>A	*ND2*	37	ATG>ATA	Met>Iso	p.M37I	non-syn	1	-
17	4615	A>G	*ND2*	49	AAC>AGC	Asn>Ser	p.N49S	non-syn	1	GU186071
18	4638	A>G	*ND2*	57	ATC>GTC	Ile>Val	p.I57V	non-syn	3	GU186072
19	4703	T>C	*ND2*	78	AAT>AAC	Asn>Asn	p.N78N	syn	1	-
20	4916	A>G	*ND2*	149	CTA>CTG	Leu>Leu	p.L149L	syn	1	-
21	4917	A>G	*ND2*	150	AAC>GAC	Asn>Asp	p.N150D	non-syn	1	-
22	4944	A>G	*ND2*	159	ATC>GTC	Ile>Val	p.I159V	non-syn	1	-
23	5033	A>G	*ND2*	188	GGA>GGG	Gly>Gly	p.G188G	syn	1	-
24	5097	A>G	*ND2*	210	ATC>GTC	Ile>Val	p.I210V	non-syn	1	GU186073
25	5186	A>T	*ND2*	239	TGA>TGT	Trp>Cys	p.W239C	non-syn	4	-
26	5252	G>A	*ND2*	261	TTG>TTA	Leu>Leu	p.L261L	syn	1	-
27	5360	C>T	*ND2*	297	ATC>ATT	Ile>Ile	p.I297I	syn	1	-
28	6011	T>C	*CO1*	36	CTT>CTC	Leu>Leu	p.L36L	syn	1	-
29	6023	G>A	*CO1*	4O	GAG>GAA	Glu>Glu	p.E40E	syn	1	-
30	6217	T>C	*CO1*	105	TTA>CTA	Leu>Leu	p.L105L	syn	1	GU186074
31	6290	C>T	*CO1*	129	TAC>TAT)	Tyr>Tyr	p.Y129Y	syn	3	-
32	6366	G>A	*CO1*	155	GTC>ATC	Val>Ile	p.V155 I	non-syn	3	-
33	6584	C>T	*CO1*	227	GAC>GAT	Asp>Asp	p.D227D	syn	1	GU186075
34	7280	C>T	*CO1*	459	TTC>TTT	Phe>Phe	p.F459F	syn	2	GU186076
35	7598	G>A	*CO2*	5	GCG>ACG	Ala>Thr	p.A5T	non-syn	1	-
36	7746	A>G	*CO2*	54	AAC>AGC	Asn>Ser	p.N54S	non-syn	1	GU186077
37	7843	A>G	*CO2*	86	ATA>ATG	Met>Met	p.M86M	syn	3	-
38	7868	C>T	*CO2*	95	CTT>TTT	Leu>Phe	p.L95F	non-syn	2	-
39	7961	T>C	*CO2*	126	TTA>CTA	Leu>Leu	p.L126L	syn	1	-
40	7962	T>C	*CO2*	126	TTA>TCA	Leu>Ser	p.L126S	non-syn	1	GU186078
41	8023	T>C	*CO2*	146	ATT>ATC	Ile>Ile	p.I 146 I	syn	1	-
42	8116	A>G	*CO2*	177	GGA>GGG	Gly>Gly	p.G177G	syn	1	GU186079
43	8136	T>C	*CO2*	184	TTC>TTT	Phe>Phe	p.F184F	syn	32	-
44	8252	G>A	*CO2*	222	GGG>GGA	Gly>Gly	p.G222G	syn	2	-
45	8346	C>T	*t RNA Lys*	-	-	-	-	-	1	GU186096
46	8396	A>G	*ATPase8*	11	ACC>GCC	Thr>Ala	p.T11A	non-syn	1	GU186091
47	8485	G>A	*ATPase8*	40	AAG>AAA	Lys>Lys	p.K40K	syn	1	GU186092
48	8502	A>G	ATPase8	46	AAT>AGT	Asn>Ser	p.N46S	non-syn	1	GU186091
49	8584	G>A	*ATPase6*	20	GCA>ACA	Ala>Thr	p.A20T	non-syn	2	-
50	8658	C>T	*ATPase6*	44	ACC>ACT	Thr>Thr	p.T44T	syn	1	GU186080
51	8684	C>T	*ATPase6*	53	ACC>ATC	Thr>Ile	p.T53I	non-syn	1	-
52	8697	G>A	*ATPase6*	57	ATG>ATA	Met>Met	p.M57M	syn	1	-
53	8701	G>A	*ATPase6*	59	GCC>ACC	Ala>Thr	p.A59T	non-syn	15	-
54	8843	T>A	*ATPase6*	106	ATC>ACC	Ile>Ile	p.I106 I	syn	2	-
55	8865	G>A	*ATPase6*	113	GTG>GTA	Val>Val	p.V113V	syn	2	-
56	8875	T>C	*ATPase6*	117	TTT>CTT	Phe>Thr	p.F117T	non-syn	1	GU186081
57	8886	G>A	*ATPase6*	120	AAG>AAA	Lys>Lys	p.K120K	syn	35	GU186095
58	8887	A>G	*ATPase6*	121	ATT>GTT	Ile>Val	p.I121V	non-syn	1	-
59	8925	A>G	*ATPase6*	133	ACA>ACG	Thr>Thr	p.T133T	syn	1	-
60	8928	T>C	*ATPase6*	138	ATC>ACC	Ile>Thr	p.I138T	non-syn	1	-
61	8943	C>T	*ATPase6*	139	CCC>CCT	Pro>Pro	p.P139P	syn	1	-
62	9064	G>A	*ATPase6*	180	GCA>ACA	Ala>Thr	p.A180T	non-syn	2	-
63	9072	A>G	*ATPase6*	183	TCA>TCG	SerSer	p.S183S	syn	1	-
64	9094	C>T	*ATPase6*	190	CTT>TTT	Leu>Phe	p.L190F	non-syn	1	-
65	9110	T>C	*ATPase6*	195	ATT>ACT	Ile>Thr	p.I195T	non-syn	1	-
66	9287	G>A	*CO3*	27	ATG>ATA	Met>Met	p.M27M	syn	1	GU186089
67	9377	G>A	*CO3*	57	TGA>TGG	Trp>Trp	p.W57W	syn	19	-
68	9540	C>T	*CO3*	112	CTA>TTA	Leu>Leu	p.L112L	syn	9	-
69	9614	A>G	*CO3*	136	GTA>GTG	Val>Val	p.V136V	syn	1	-
70	9698	T>C	*CO3*	164	CTT>CTC	Leu>Leu	p.L164L	syn	1	-
71	9716	T>C	*CO3*	170	GGT>GGC	Gly>Gly	p.G170G	syn	1	-
72	9767	C>T	*CO3*	183	ACC>ACT	Thr>Thr	p.T187T	syn	1	-
73	9768	T>C	*CO3*	184	TCT>TCC	Ser>Ser	p.S184S	syn	1	-
74	9967	G>A	*CO3*	254	GTC>ATC	Val>Ile	p.V254I	non-syn	1	GU186090
75	10142	C>T	*ND3*	28	AAC>AAT	Asn>Asn	p.N28N	syn	1	-
76	10181	C>T	*ND3*	41	TTC>TTT	Phe>Phe	p.F41F	syn	1	-
77	10365	G>C	*ND3*	103	GCC>ACC	Ala>Thr	p.A103T	non-syn	1	-
78	10398	G>A	*ND3*	114	GCC>ACC	Ala>Thr	p.A114T	non-syn	16	-
79	10400	C>T	*ND3*	114	GCC>GCT	Ala>Ala	p.A114A	syn	12	-
80	10410	T>A	*tRNA Arg*	-	-	-	-	-	1	-
81	10463	T>C	*tRNA Arg*	-	-	-	-	-	1	-
82	10490	T>C	*ND4L*	7	AAT>AAC	Asn>Asn	p.N7N	syn	1	GU186094
83	10550	A>G	*ND4L*	27	ATA>ATG	Met>Met	p.M27M	syn	1	-
84	11467	A>G	*ND4*	236	TTA>TTG	Leu>Leu	p.L236L	syn	2	-
85	11719	A>G	*ND4*	320	GGA>GGG	Gly>Gly	p.G320G	syn	2	-
86	12007	G>A	*ND4*	416	TGG>TGA	Trp>Trp	p.W416W	syn	5	-
87	12031	C>T	*ND4*	424	AAC>AAT	Asn>Asn	p.N424N	syn	1	GU186088
88	12097	C>T	*ND4*	446	CTC>CTT	Leu>Leu	p.L446L	syn	2	-
89	12106	C>T	*ND4*	449	CTC>CTT	Leu>Leu	p.L449L	syn	1	-
90	12130	T>C	*ND4*	457	TTT>TTC	Phe>Phe	p.F457F	syn	1	GU186087
91	12308	A>G	*tRNA Leu*	-	-	-	-	-	3	-
92	12372	G>A	*ND5*	12	CTG>CTA	Leu>Leu	p.L12L	syn	4	-
93	12477	T>C	*ND5*	47	AGT>AGC	Ser>Ser	p.S47S	syn	1	-
94	12498	C>T	*ND5*	54	TTC>TTT	Phe>Phe	p.F54F	syn	2	-
95	12561	G>A	*ND5*	75	CAG>CAA	Gln>Gln	p.Q75Q	syn	1	-
96	12681	T>C	*ND5*	115	AAT>AAC	Asn>Asn	p.N115N	syn	1	-
97	12705	T>C	*ND5*	123	ATT>ATC	Ile>Ile	p.I123 I	syn	10	-
98	12793	T>C	*ND5*	153	TTG>CTG	Leu>Leu	p.L153L	syn	1	GU186084
99	12810	A>G	*ND5*	158	TGA>TGG	Trp>Trp	p.W158W	syn	1	-
100	12849	A>T	*ND5*	171	GCA>GCT	Ala>Ala	p.A171A	syn	8	GU186085
101	12879	T>C	*ND5*	181	GGT>GGC	Gly>Gly	p.G181G	syn	1	-
102	12906	C>T	*ND5*	190	ATC>ATT	Ile>Ile	p.I190I	syn	1	GU186082
103	13020	T>C	*ND5*	228	GGT>GGC	Gly>Gly	p.G228G	syn	1	-
104	13135	G>A	*ND5*	267	GCA>ACA	Ala>Thr	p.A267T	non-syn	1	-
105	13138	G>A	*ND5*	268	GAA>AAA	Glu>Lys	p.E268K	non-syn	20	GU186083
106	13188	C>T	*ND5*	284	ACC>ACT	Thr>Thr	p.T284T	syn	1	-
107	13194	G>A	*ND5*	286	CTG>CTA	Leu>Leu	p.L286L	syn	1	-
108.	13215	T>C	*ND5*	293	CTT>CTC	Leu>Leu	p.L293L	syn	1	-
109	13368	G>A	*ND5*	344	GGG>GGA	Gly>Gly	p.G344G	syn	1	-
110	13500	T>C	*ND5*	388	GGT>GGC	Gly>Gly	p.G388G	syn	1	-
111	13539	A>G	*ND5*	401	ATA>ATG	Met>Met	p.M401M	syn	2	-
112	13542	A>G	*ND5*	402	TCA>TCG	Ser>Ser	p.S402S	syn	1	-
113	13651	A>G	*ND5*	439	ACC>GCC	Thr>Ala	p.T439A	non-syn	1	-
114	13656	T>C	*ND5*	440	CTT>CTC	Leu>Leu	p.L440L	syn	4	-
115	13708	G>A	*ND5*	458	GCA>ACA	Ala>Thr	p.A458T	non-syn	1	-
116	13768	T>C	*ND5*	478	TTC>CTC	Phe>Leu	p.F478L	non-syn	1	GU186086
117	14000	T>A	*ND5*	555	CCT>CCA	Leu>Pro	p.L555P	non-syn	2	-
118	14022	A>G	*ND5*	562	TTA>TTG	Leu>Leu	p.L562L	syn	1	-
119	14935	T>C	*CYB*	63	TTT>TTC	Phe>Phe	p.F63F	syn	1	-
120	15038	A>G	*CYB*	98	ATC>GTC	Ile>Val	p.I98V	non-syn	1	-
121	15043	G>A	*CYB*	99	GGG>GGA	Gly>Gly	p.G99G	syn	12	-
122	15061	A>G	*CYB*	105	GGA>GGG	Gly>Gly	p.G105G	syn	1	-
123	15097	T>C	*CYB*	117	ATT>ATC	Ile>Ile	p.I117I	syn	1	-
124	15148	G>A	*CYB*	134	CCG>CCA	Pro>Pro	p.P134P	syn	3	-
125	15301	G>A	*CYB*	185	TTG>TTA	Leu>Leu	p.L185L	syn	16	-
126	15317	G>A	*CYB*	191	GCC>ACC	Ala>Thr	p.A191T	non-syn	1	-
127	15452	C>A	*CYB*	236	CTT>ATT	Leu>Ile	p.L236 I	non-syn	2	-
128	15607	A>G	*CYB*	287	AAA>AAG	Lys>Lys	p.K287K	syn	1	-
129	15670	T>C	*CYB*	308	CAT>CAC	His>His	p.H308H	syn	1	-
130	15683	A>G	*CYB*	312	CAA>CAG	Gln>Gln	p.Q312Q	syn	2	GU186093
131	At 15928	G>A	*t RNA Thr*	-	-	-	-	-	1	-
132	At 15930	G>A	*t RNA Thr*	-	-	-	-	-	1	-

Key: A – Adenine; T – Thymine; G- Guanine; C- Cytosine; Syn- synonymous; Non-syn- non-synonymous; *- novel change; *ND1*- NADH dehydrogenase subunit 1; *ND2*- NADH dehydrogenase subunit 2; *ND3*- NADH dehydrogenase subunit 3; *ND4*- NADH dehydrogenase subunit 4; *ND4L*- NADH dehydrogenase subunit 4L; *ND5*- NADH dehydrogenase subunit 5; *CO1*- cytochrome c oxidase I; *CO2*- cytochrome c oxidase II; *CO3*- cytochrome c oxidase III; *ATPase6*- ATP synthase subunit a (F-ATPase protein 6); *ATPase8*- ATP synthase protein 8; *CYB*- cytochrome B; *tRNA*- transfer ribo nucleic acid; *rRNA*- ribosomal ribonucleic acid.

**Table 2:** Mitochondrial DNA variations observed in controls.

**Sample number**	**Genomic position**	**Base change**	**Gene/ Location**	**Amino acid position**	**Codon change**	**Amino acid change**	**Change in protein**	**Syn/ Non-syn**	**GenBank accession number if novel**
1	3591	G>A	*ND1*	95	CTG>CTA	Thr>Thr	p.T95T	syn	-
2	3915	G>A	*ND1*	203	GGG>GGA	Gly>Gly	p.G203G	syn	-
3	3918	G>A	*ND1*	204	GAG>GAA	Glu>Glu	p.E204E	syn	-
4	3933	A>G	*ND1*	209	TCA>TCG	Ser>Ser	p.S209S	syn	GU397544
5	3970	C>T	*ND1*	222	CTA>TTA	Leu>Leu	p.L222L	syn	-
6	3996	C>T	*ND1*	230	AAC>AAT	Asn>Asn	p.N230N	syn	-
7	4029	C>A	*ND1*	241	ATC>ATA	Ile>Ile	p.I241I	syn	GU397545
8	4093	A>G	*ND1*	263	ACC>GCC	Thr>Ala	p.T263A	non-syn	-
9	4793	A>G	*ND2*	108	ATA>ATG	Met>Met	p.M108M	syn	-
10	4852	T>A	*ND2*	128	CTG>CAG	Leu>Gln	p.L128Q	non-syn	GU397533
11	5186	A>T	*ND2*	239	TGA>TGT	Trp>Cys	p.W239C	non-syn	-
12	5348	C>T	*ND2*	293	TAC>TAT	Tyr>Tyr	p.Y293Y	syn	-
13	5351	A>G	*ND2*	294	CTA>CTG	Leu>Leu	p.L294L	syn	-
14	6305	G>A	*CO1*	134	GGG>GGA	Gly>Gly	p.G134G	syn	-
15	6719	T>C	*CO1*	272	GGT>GGC	Gly>Gly	p.G272G	syn	-
16	6962	G>A	*CO1*	353	CTG>CTA	Thr>Thr	p.T353T	syn	-
17	7316	G>A	*CO1*	471	ATG>ATA	Met>Met	p.M471M	syn	–
18	7738	T>C	*CO2*	51	ACT>ACC	Thr>Thr	p.T51T	syn	-
19	7762	G>A	*CO2*	59	CAG>CAA	Gln>Gln	p.Q59Q	syn	-
20	8143	T>C	*CO2*	186	GCT>GCC	Ala>Ala	p.A186A	syn	GU397549
21	8251	G>A	*CO2*	222	GGG>GGA	Gly>Gly	p.G222G	syn	-
22	8503	T>G	*ATP8*	46	AAT>AAG	Asp>Lys	p.N46K	non-syn	GU397551
23	8584	G>A	*ATP6*	20	GCA>ACA	Ala>Thr	p.A20T	non-syn	-
24	8594	T>C	*ATP6*	23	ATC>ACC	Ile>Thr	p.I23T	non-syn	-
25	8650	C>T	*ATP6*	42	CTA>TTA	Leu>Leu	p.L42L	syn	-
26	8718	A>G	*ATP6*	64	AAA>AAG	Lys>Lys	p.K64K	syn	-
27	8812	A>G	*ATP6*	96	ACC>GCC	Thr>Ala	p.T96A	non-syn	-
28	8886	G>A	*ATP6*	120	AAG>AAA	Lys>Lys	p.K120K	syn	GU397552
29	8925	A>G	*ATP6*	133	ACA>ACG	Thr>Thr	p.T133T	syn	-
30	10310	G>A	*ND3*	84	CTG>CTA	Thr>Thr	p.T84T	syn	-
31	10609	T>C	*ND4L*	47	ATA>ACA	Met>Thr	p.M47T	non-syn	-
32	11467	A>G	*ND4*	236	TTA>TTG	Leu>Leu	p.L236L	syn	-
33	11914	G>A	*ND4*	385	ACG>ACA	Thr>Thr	p.T385T	syn	-
34	12007	G>A	*ND4*	416	TGG>TGA	Trp>Trp	p.W416W	syn	-
35	12073	C>T	*ND4*	438	TTC>TTT	Phe>Phe	p.F438F	syn	GU397542
36	12107	C>T	*ND4*	449	CTC>CTT	Thr>Thr	p.T449T	syn	GU397543
37	12133	C>T	*ND4*	458	TCC>TCT	Ser>Ser	p.S458S	syn	-
38	12372	G>A	*ND5*	12	CTG>CTA	Tyr>Tyr	p.T12T	syn	-
39	12373	A>G	*ND5*	13	ACT>GCT	Thr>Ala	p.T13A	non-syn	-
40	12406	G>A	*ND5*	24	GTT>ATT	Val>Ile	p.V24I	non-syn	-
41	12486	C>T	*ND5*	50	CCC>CCT	Pro>Pro	p.P50P	syn	GU397534
42	12498	C>T	*ND5*	54	TTC>TTT	Phe>Phe	p.F54F	syn	GU397538
43	12561	G>A	*ND5*	75	CAG>CAA	Gln>Gln	p.Q75Q	syn	-
44	13204	G>A	*ND5*	290	GTC>ATC	Val>Ile	p.V290I	non-syn	GU397535
45	13299	A>G	*ND5*	321	CAA>CAG	Gln>Gln	p.Q321Q	syn	-
46	13676	A>G	*ND5*	447	AAC>AGC	Asn>Ser	p.N447S	non-syn	GU397540
47	13731	A>G	*ND5*	465	GGA>GGG	Gly>Gly	p.G465G	syn	-
48	13860	C>T	*ND5*	490	GCC>GCT	Ala>Ala	p.A490A	syn	GU397541
49	14058	C>T	*ND5*	574	TCC>TCT	Ser>Ser	p.S574S	syn	-
50	14783	T>C	*CYB*	13	TTA>CTA	Leu>Leu	p.L13L	syn	-
51	14872	C>T	*CYB*	42	ATC>ATT	Ile>Ile	p.I42I	syn	-
52	15119	G>A	*CYB*	125	GCA>ACA	Ala>Thr	p.A125T	non-syn	-
53	15172	G>A	*CYB*	142	GGG>GGA	Gly>Gly	p.G142G	syn	-
54	15217	G>A	*CYB*	157	GGG>GGA	Gly>Gly	p.G157G	syn	-
55	15385	C>T	*CYB*	213	TCC>TCT	Ser>Ser	p.S213S	syn	-
56	15431	G>A	*CYB*	229	GCC>ACC	Ala>Thr	p.A229T	non-syn	-
57	15484	A>G	*CYB*	246	TCA>TCG	Ser>Ser	p.S246S	syn	GU397547
58	15670	T>C	*CYB*	308	CAT>CAC	His>His	p.H308H	syn	-

Key: A – Adenine; T – Thymine; G- Guanine; C- Cytosine; Syn- synonymous; Non-syn- non-synonymous; ND1- NADH dehydrogenase subunit 1; ND2- NADH dehydrogenase subunit 2; ND3- NADH dehydrogenase subunit 3; ND4- NADH dehydrogenase subunit 4; ND4L- NADH dehydrogenase subunit 4L; ND5- NADH dehydrogenase subunit 5; CO1- cytochrome c oxidase I; CO2- cytochrome c oxidase II; CO3- cytochrome c oxidase III; ATPase6- ATP synthase subunit a (F-ATPase protein 6); ATPase8- ATP synthase protein 8; CYB- cytochrome B.

Two non-synonymous changes (p.W239C in *ND2* and p.A20T in *ATPase6*) were present both in cases as well as controls. The remaining 40 non-synonymous changes were limited to PCG cases only. All novel variations from patients and controls were submitted to the GenBank database and accession numbers were obtained (Table 1 and Table 2).

SIFT and PolyPhen analysis of all non-synonymous changes from cases and controls revealed five pathogenic changes (p.P2L, p.I10T, p.M31T in ND1 protein and p.M37I, p.W239C in ND2 protein). Eight patients (22.85%) were positive for either of these pathologic mtDNA nucleotide changes. Clinical features of patients and mtDNA variations identified in this study have been tabulated (Table 3).

**Table 3:** Mitochondrial DNA variations and clinical features of primary congenital glaucoma patients.

	**Sequence variations in different mitochondrial genes**															
**Patient number**	***ND1***	***ND2***	***CO1***	***CO2***	***ATPase8***	***ATPase6***	***CO3***	***ND3***	***ND4L***	***ND4***	***ND5***	***CYB***	**Other changes**	**Last cup disc ratio (OS/OD) at presentation**	**Corneal diameter (mm) OS/OD**	**IOP (mmHg) OS/OD**
1	–	–	p.Y129Y; p.F459F	p.M86M; p.L95F	–	p.A20T; p.A59T	p.W57W; p.L112L	p.A114T	-	–	p.I123I; p.E268K; p.M401M; p.L555P	p.P134P; p.L185L	Ins. of T at 2790_91 (16sRNA)	Total cupping	15x15/15x15.5	40/30
2	–	–	p.Y129Y; p.F459F	p.M86M; p.L95F	–	p.A20T; p.A59T	p.W57W; p.L112L	p.A114T	-	–	p.I123I; p.E268K; p.M401M; p.L555P	p.P134P; p.L185L	Ins. of T at 2790_91 (16sRNA)	0.8:1/0.8:1	13x13/13x13	36/40
3	p.M31T		p.V155 I		–	p.V113V	p.W57W	p.A114A	-	p.W416W	p.E268K	p.G99G	-	Hazy MEDIA	15x14/15x15	26/38
4	–	–	p.D227D	G222G	–	I195T	W57W	A114A	-	p.W416W	p. E268K	p.A191T; p.Q312Q	-	0.4:1/0.4:1	12x11/12x12	22/24
5	–	–	–	p.N54S	–	p.A59T	p.W57W; p.L112L	p.A114T	-		p.I190I; p.E268K	p.G99G; p.L185L	-	0.7:1/0.7:1	14.5x15/15x15	28/28
6	p.Y304H; p.M307V	p.N150T	–	–		p.M57M	p.W57W; p.L112L	p.A114T	-	–	p. I123I; p.E268K; p.G344G	p.L236I; p.K287Q	-	0.5:1/0.5:1	12x13/12x13	22/23
7	–	–	–	p.I146I; p.F184F	–	p.A59T; p.P139P	p.M27M; p.W57W; p.L112L; p.T187T	p.A114T	-	p.L236L	p.I123I; p.E268K	p.F63F; p.G99G; p.G105G; p.L185L	A>G at 12308 tRNA Leu	0.4:1/0.4:1	12x13/11x11.5	38/14
8	–	–	–	p.G177G	–	–	p.W57W	p.A114A	-	–	p.Q75Q; p.E268K	p.G99G	G>A at 15928 (t RNA Thr)	Hazy media	NA	23/25
9	p.L174L; p.S205S	–	–	–	–	–	p.W57W	p.A114A	-	–	p.I123I; p.V172I; p.E268K; p.L293L	p.G99G	C>T at 8346 (t RNA Lys)	NA	12x12/12x12	22/22
10	p.V113V; p.T145T	p.S11S; p.I297I		L126S; F184F		p.I106I; p.T53 I	p.W57W; p.L112L	p.N28N; p.A114T	-	p.L236L	p.I123I; p.E268K; p.G388G	–	A>G at 12308 tRNA Leu	NA	NA	23/24
11	–	p.W239C	p.L105T	–	–	p.A59T; p.L190F	p.W57W; p.L112L; p.V136V	p.A114T	-	p.L449L	p.I123I; p.L153L; p.E268K; p.L286L	p.L185L	G>A at 2707 (16sRNA); A>G at 12308 tRNA Leu; G>A at 15930 tRNA Arg	NA	14x14/14x15	26/22
12	p.P2L	p.N49S	–	–	–	A59T; I106I	–	p.A114T	-	p.G320G	p.N115N; p.I123I; p.E268K; p.L440L; p.A458T; p.F478L	p.L185L	-	NA	14x14.5/14x14.4	32/32
13	p.M31T	–	p.V155 I	–	–	p.V113V	p.W57W	p.A114A	-	p.W416W	p.A171A; E268K	p.G99G	-	Hazy media	14x14/14x14	31/30
14	–	–	–	–	–	p.S183S	p.W57W	p.A114A	-	p.W416W	p.A171A; p.E268K; p.T284T	p.G99G	-	Hazy media	NA	25/24
15	–	p.L261L	–	p.L126L	p.T11A; p.N46S	–	p.W57W; p.S184S	p.A114A	-	–	p.A171A; p.E268K	p.G99G; p.H308H	-	0.6:1/0.6:1	14.5x14/13.5x13	32/32
16	–	–	–	–	p.K40K	–	p.W57W	p.A114A	-	p.W416W	p.F54F; p.A171A; p.A267T; p.E268K	p.G99G	-	0.4:1/0.5:1	11x11/12x12.5	18/26
17	p.S205S	–	–	–	–	p.T44T	p.W57W	p.A114A	-	p.F457F	S47S; A171A; G181G; E268K	p.G99G	-	NA	14x14/14x14.5	30/28
18	–	–	–	–	–	p.A59T	p.W57W; p.L112L	p.A114T	-	p.G320G	p.I123I; p.A171A; p.E268K	p.L185L; p.Q312Q	-	NA	14.5x14/14x14	20/20
19	–	–	p.V155 I	p.A5T	–	p.F17T; p.A180T	p.W57W	p.A103T; p.A114A	-	–	p.A171A; p.G228G; p.E268K	p.G99G	-	0.5:1 1	12x12.5/12x12	22/22
20	p.I10T	–	p.Y129Y	p.M86M	–	p.A59T	p.W57W; p.L112L	p.A114T	-	–	p.I123I; p.V172I	p.I98V; p.P134P; p.L185L	-	Hazy media	12x13/13x13	18/37
21	–	p.I59V	–	–	–	p.K120K	–	–	-	p.L446L	–	p.L185L	-	Hazy media	15x16/11.5x12	32/15
22	–	–	–	p.G222G	–	p.K120K		p.A114A	-	-	–		-	0.7:1/0.5:1	15x15/16x16	28/28
23	p.K58K	p.V31A		–	–	p.A59T; p.K120K	p.L164L; p.G170G	p.A114T	p.M27M	-	p.L12L	p.L185L	-	Hazy media	14x15/14x15	34/36
24	–		–	–	–	p.A59T; p.K120K; p.P134P	-		-	-	–	p.L185L; p.L236I	T>A at 10411 (t RNA Arg)	0.7:1/0.7:1	NA	32/10
25	–	p.I57V; p.W239C	–	–	–	p.A59T; p.K120K	-	p.A114T	-	-	p.L12L; p.L440L	–	-	NA	14x15/14x15	22/22
26	–	p.I57V; p.W239C	–	–	–	p.A59T; p.K120K	-	p.A114T	-	-	p.L12L; p.L440L	–	-	Hazy media	13x13.5/15x14.5	22/22
27	–	p.L149L; p.G188G	–	–	–	p.K120K	-	p.A114A	-	-	p.T439A	p.L185L	-	0.8:1/0.8:1	13x13.5/13x13.5	20/20
28	–	p.M37I; p.N78N	–	–	–	p.K120K	-	p.A114A	-	-	–	p.G99G	-	hazy media	14x14/14x14	22/24
29	–	–	p.E40E	–	–	p.A59T; p.K120K	-	p.A114T	p.N7N	-	–	–	-	NA	14x14/11x11	18/22
30	p.A4T	–	–	–	–	p.K120K ; p.I121V	V254I	p.F41F; p.A114T	-	p.N424N	–	p.L185L	-	Hazy media	corneal ulcer/14x14	18/20
31	–	p.I210V	p.L36L	–	–	p.K120K	-	p.A114A	-	-	–	p.I117I	-	Hazy media	12x10/12x12.5	40/26
32	–	p.I57V; p.W239C	–	–	–	p.A59T; p.K120K; p.A180T	-	p.A114T	-	-	p.L12L	p.L185L	-	0.8:1 1	12x12/12x12	22/26
33	–	–	–	–	–	p.K120K	-	–	-	-	–	p.L185L	-	0.8:1/0.9:1	14x14/14x14	26/24
34	–	–	–	-	-	p.K120K; p.T133T	-	p.A114A	-	-	p.S402S	–	-	0.5:1/0.5:1	12x12/12x12.5	20/22
35	p.K58K	–	–	–	–	p.K120K	-	p.A114A	-	p.L446L	–	–	–	0.5:1/0.3:1	13x13/13x13.5	24/16

Key: NA- not available; OD- right eye; OS- left eye; OU- both eyes; mmHg- milimeter of mercury; IOP- intra ocular pressure; Syn- synonymous; Non-syn- non-synonymous; - novel change; *ND1*- NADH dehydrogenase subunit 1; *ND2*- NADH dehydrogenase subunit 2; *ND3*- NADH dehydrogenase subunit 3; *ND4*- NADH dehydrogenase subunit 4; *ND4L*- NADH dehydrogenase subunit 4L; *ND5*- NADH dehydrogenase subunit 5; *CO1*- cytochrome c oxidase I; *CO2*- cytochrome c oxidase II; *CO3*- cytochrome c oxidase III; *ATPase6*- ATP synthase subunit a (F-ATPase protein 6) ; *ATPase8*- ATP synthase protein 8; *CYB*- cytochrome B; tRNA- transfer ribo nucleic acid.

## Discussion

**Figure 2:**
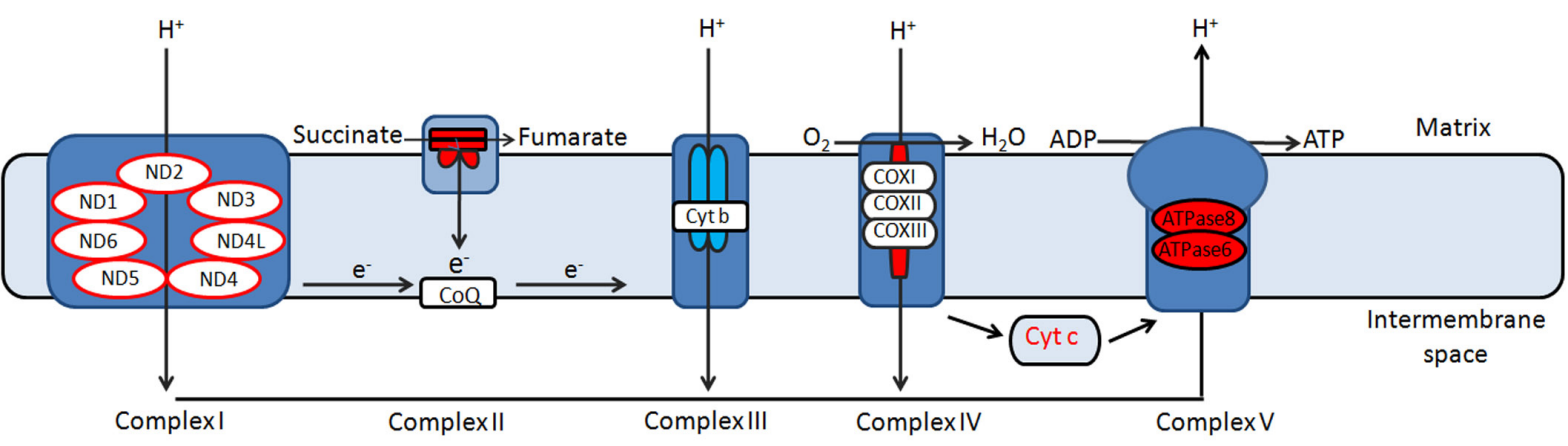
Schematic representation of components of the OXPHOS pathway localized in inner mitochondrial membrane.

The human mtDNA is a 16,569-base pair double-stranded, compact, circular molecule which lacks histones and is without introns. MtDNA has several overlapping genes and incomplete termination codons. It contains 37 genes which regulate oxidative phosphorylation (OXPHOS). Of these, 24 are needed for mtDNA translation (2 rRNAs [rRNAs] and 22 tRNAs [tRNAs]), and 13 encode subunits of the respiratory chain: seven subunits of complex I (ND1, 2, 3, 4, 4L, 5, and 6 [ND stands for NADH dehydrogenase]), one subunit of complex III (cytochrome b), three subunits of cytochrome c oxidase (COX I, II, and III), and two subunits of ATP synthase (ATPase6 and ATPase8; Figure 2).

MtDNA mutates 10 times more frequently as compared to nuclear DNA due to its proximity to the electron transport chain (ETC) and lack of histones and other protective proteins and has very basic repair mechanism [23]. Mitochondria are essential for ATP production by OXPHOS and are susceptible to oxidative damage because reactive oxygen species (ROS) damage mitochondrial enzymes directly and alter mitochondrial membrane permeability leading to cell death [24]. Most studies suggest that the majority of intracellular ROS produced by non-phagocytic cells are derived from mitochondria [25,26]. Thus mitochondria are both source and target of free radicals.

Several human diseases have been associated with mtDNA mutations, indicating that dysfunction of the components of oxidative phosphorylation encoded by the mitochondrial genome can be deleterious [27]. Abnormalities in mtDNA have proven to be associated with leber’s hereditary optic neuropathy (LHON) [28], POAG, pseudoexfoliation glaucoma (PEG), primary angle closure glaucoma (PACG), other spontaneous optic neuropathies [11,13,14,29], and male infertility [15].

In this study we screened 35 PCG cases for mtDNA variations. We found 42 non-synonymous mtDNA variations in PCG patients in different mitochondrial genes. The highest number of nucleotide variations were recorded in complex I, followed by complex IV and then complex V. Eight patients (22.85%) were found to be positive for pathogenic changes while in PEG patients this was 10.30% [14].

Complex I is responsible for pumping of protons (H^+^) from the matrix to the inter-membrane space in association with complex III and IV. Although the mitochondrial ETC is very effective in the reduction of oxygen to water, there is a constant “leak” of electrons from the ETC to oxygen and this results in the formation of superoxide anions. It is generally agreed that there are two main sites in the respiratory chain where superoxide anions are generated viz. complex I and complex III [30,31]. Dismutation of superoxide anions produces hydrogen peroxide as a secondary product, and in the presence of transition metals this can be converted to a highly reactive hydroxyl radical that can readily oxidize proteins, lipids, carbohydrates, DNA, and RNA [32]. Fifty percent nucleotide variations identified in our study were in complex I.

SIFT and PolyPhen analysis of missense changes showed that p.P2L, p.I10T, and p.M31T in ND1 protein and p.M37I and p.W239C in ND2 protein were deleterious to protein function. PHAT (predicted hydrophobic and transmembrane) score difference of p.P2L and p.I10T was >2 and PSIC score of p.M31T was >2. PSIC score of p.M37I and W239C was >2 and >1.5, respectively. All these changes (p.P2L, p.I10T, and p.M31T in *ND1* and p.M37I and p.W239C in *ND2*) had SIFT scores <0.05 and were predicted to be deleterious. Pathogenic variants p.P2L, p.I10T, and p.M31T were present in 3 cases (1 case each) while p.M37I and p.W239C were present in one and four cases, respectively. However, frequency of the pathogenic variants (p.P2L, p.I10T, and p.M31T in *ND1* and p.M37I and p.W239C in *ND2*) was not found to be statistically significant (p value >0.05) in our study population.

Recent studies have shown that G4580A (p.M37I) in *ND2* and G10398A (p.A114T) in *ND3* are associated with an increase in production of ROS due to altered complex I function [33-35]. G4580A (p.M37I) was present in 1 patient and G10398A (p.A114T) in 16 patients in our study. The frequency of G10398A (p.A114T) alteration was found to be statistically significant (p value <0.001) in our study population. Twenty patients (57.14%) had changes associated with elevated ROS production. It has been reported that alterations in mitochondrial complex I causes cytochrome c oxidase deficiency and OS [36]. Pathogenic mutations in ND genes have been reported in POAG, PACG, and PEG [11,13,14].

In this study we found mtDNA sequence changes which were different from other types of glaucoma. When compared between cases and controls, frequency of non-synonymous sequence variations in *ND2* and *ND3* were found to be statistically significant (p value <0.05; Table 4). Point mutations in *ND1*, *ND4*, and *ND6* have been reported in association with LHON [37,38]. Moreover, mutations in complex I genes are also associated with Leigh syndrome, mitochondrial encephalomyopathy, lactic acidosis stroke-like episodes (MELAS), and infertility [15,39-41].

**Table 4:** With p-value and Relative risk at 95% confidence interval by using Pearson χ^2^/Fisher’s exact test for non-synonymous sequence variations in different mitochondrial genes in PCG and controls.

**Gene name**	**Cases (n=35)**	**Controls (n=40)**	**p-value**	**Relative risk at 95% confidence interval**
*ND1*	6 (17.14%)	2 (5.0%)	0.136	1.73 (1.07–2.81)
*ND2*	10 (28.57%)	2 (5.0%)	0.005	2.10 (1.41–3.12)
*ND3*	17 (48.60%)	0 (0%)	<0.001	3.22 (2.19–4.72)
*ND4*	0 (0%)	0 (0%)	—-	—–
*ND4L*	0 (0%)	2 (5.0%)	0.495	1.92 (1.54–2.39)
*ND5*	21 (60.00%)	4 (10.0%)	<0.001	3.00 (1.86–4.83)
*CO1*	4 (11.42%)	0 (0%)	0.043	2.29 (1.75–2.98)
*CO2*	5 (14.30%)	0 (0%)	0.019	2.33 (1.78–3.05)
*CO3*	1 (2.90%)	0 (0%)	0.467	2.17 (1.70–2.78)
*CYB*	4 (11.42%)	8 (20.00%)	0.360	0.57 (0.18–1.73)
*ATPase6*	18 (51.42%)	7 (17.50%)	0.002	2.12 (1.34–3.34)
*ATPase8*	1 (2.85%)	1 (2.5)	1.00	1.07 (0.26–4.40)
Others	8 (22.85%)	0 (0%)	0.001	2.48 (1.85–3.32)

Abbrevations: *ND1*- NADH dehydrogenase subunit 1; *ND2*- NADH dehydrogenase subunit 2; *ND3*- NADH dehydrogenase subunit 3; *ND4*- NADH dehydrogenase subunit 4; *ND4L*- NADH dehydrogenase subunit 4L; *ND5*- NADH dehydrogenase subunit 5; *CO1*- cytochrome c oxidase I; *CO2*- cytochrome c oxidase II; *CO3*- cytochrome c oxidase III; *ATPase6*- ATP synthase subunit a (F-ATPase protein 6); *ATPase8*- ATP synthase protein 8; *CYB*- cytochrome B; tRNA- transfer ribo nucleic acid; rRNA- ribosomal ribonucleic acid.

Cytochrome c oxidase (COX or complex IV), the terminal enzyme of the respiratory chain (RC) catalyzes the reduction of molecular oxygen by reduced cytochrome c. This complex is composed of 13 subunits [42]. Twenty six variations (19.69%) identified in this study were present in complex IV of which six were non-synonymous. Frequency of non-synonymous sequence variations in *COI* and *COII* in PCG cases were found to be statistically significant (p value <0.05; Table 4). Human diseases associated with COX mutations include POAG, PACG, PEG, and Leigh syndrome [11,13,14,43]. The frequency of non-synonymous sequence variations in *CYB* was not found to be statistically significant (p value >0.05; Table 4).

In the current study, 15.15% mtDNA variations (20/132) were observed in complex V (*ATPase6* and *ATPase8*). Mutations in *ATPase6* have been reported in POAG, PACG, PEG, neuropathy, ataxia, retinitis pigmentosa (NARP), and mitochondrial DNA-associated Leigh Syndrome (MILS) patients [11,13,14,44,45]. Mitochondrial variations in *ATPase6* and *ATPase8* have been reported in spinocerebellar ataxias [46].

The A12308G variation in tRNA leu gene is also associated with increased ROS production [34] and this variation was detected in three patients in this study. However, the frequency of the A12308G variation and non-synonymous variations in *ATPase8* was not found to be statistically significant (p value >0.05) while that of non-synonymous variations in *ATPase6* and others (16s RNA, tRNA), as shown in Table 4, were statistically significant (p value <0.05).

**Figure 3:**
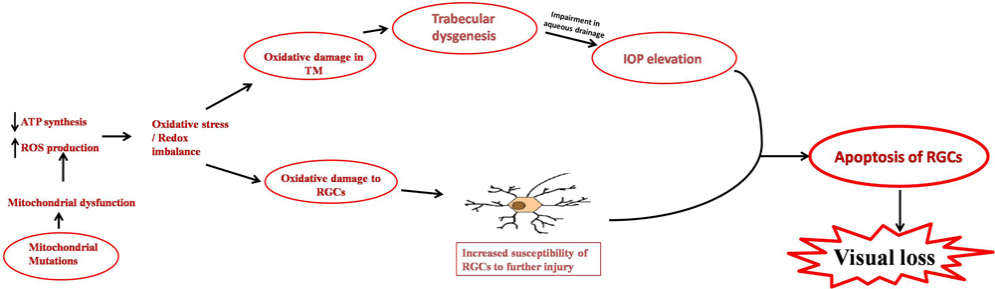
Possible role of mitochondrial sequence variations in trabecular-dysgenesis and RGC cell death in primary congenital glaucoma. Abbrevations: RGCs- Retinal ganglion cells; TM- trabecular meshwork; ROS- reactive oxygen species; ATP- adenosine tri phosphate; IOP- intra ocular pressure.

Non-synonymous mitochondrial variations adversely affect oxidative phosphorylation resulting in decreased mitochondrial respiration and increased free radical (FR) production [47]. Thus, we hypothesize that mtDNA variations with resultant lower ATP levels may impair growth, development, and differentiation of TM and result in trabecular-dysgenesis (Figure 3); a characteristic feature of PCG. Trabecular-dysgenesis leads to impairment in aqueous drainage hence causes elevation in IOP. ROS levels may increase to supraphysiological levels in TM endothelial cells and due to low ATP levels these cells are unable to eliminate the reactive oxygen intermediates (ROI). MtDNA mutations are also associated with optic neuropathies like LHON [38], NARP [48,49] or Leigh syndrome [50]. The mechanisms by which mitochondrial abnormalities may place the optic nerve at risk remain uncertain [51].

Distribution of high number of mitochondria in the optic nerve head reflects the high energy requirement of the human optic nerve head. Neurons, because of their high energy requirement, are heavily dependent on mitochondria for survival [52]. Mitochondria not only constitute an energy-generating system, but are also critically involved in calcium signaling and apoptosis. Mitochondrial function is impaired by mutations in mitochondrial and nuclear DNA, chemical insults to components of the electron transport chain, and a lack of substrates such as oxygen. The latter is relevant to tissue hypoxia that is believed to be present in the glaucomatous retina and optic nerve head either primarily or secondary to elevated IOP. Any malfunction of the mitochondrial electron transport chain results in excessive generation of free radicals and low ATP production. In our study we identified 50.00% variations in complex I, 9.02% in complex III, 19.54% in complex IV, and 15.03% in complex V. The presence of primary LHON mutations has been investigated previously in normal tension glaucoma and POAG [12,53] but not in PCG. None of PCG cases had primary LHON mutations (3460G>A, 11778G>A, 14484T>C) in the current study.

It has already been reported that OS leads to oxidative damage to cellular macromolecules such as mitochondrial and nuclear DNA, proteins, and lipids, along with energy depletion and a local dysregulation of calcium homeostasis, resulting in neuronal degeneration [54]. OS is the underlying etiology in several ocular diseases [11,54-59] and plays an essential role in early retinal ischemic injury [60] and glaucoma pathogenesis [61,62]. Mitochondrial dysfunction leads to RGC death through caspase-dependent and caspase-independent pathways initiated by the loss of mitochondrial membrane potential, release of cell death mediators and OS [54]. Glaucomatous eyes have a significant increase in OS and decreased antioxidant activity [62]. Seppet et al. [63] reported that OS is a critical factor in injury to anterior segment of eye. OS has also been reported to induce human trabecular meshwork degenerative changes that favor increased intraocular pressure [64]. Oxidative DNA damage is significantly increased in the trabecular-meshwork (TM) of glaucomatous patients compared to controls [11]. The pathogenic role of ROS in glaucoma is supported by various experimental findings, including (a) resistance to aqueous humor outflow is increased by hydrogen peroxide by inducing TM degeneration and (b) intraocular-pressure increase and severity of visual loss in glaucoma patients parallel to the amount of oxidative DNA damage affecting TM [11]. Oxidative damage constitutes an important pathogenic step triggering TM degeneration which results in intraocular hypertension. OS thus affects both TM and retinal ganglion cells, and may be involved in the neuronal cell death affecting the optic nerve in glaucoma (Figure 3).

Further evidence of oxidative damage in trabecular meshwork in glaucoma [57] and neural degeneration is that many retinal proteins exhibit oxidative modifications in experimental glaucoma [65], and may lead to important structural and functional alterations. Thus, the structure and function of mitochondria are critical determinants of endothelial cells and neuronal health. Essentially, once the mitochondrial lipid bilayer is compromised after the mitochondrial translocation of Bcl-2–associated X protein (Bax), cell death is inevitable, because of already triggered events.

It has been established that pathogenic mitochondrial mutations can cause mitochondrial dysfunction and enhance OS, which in turn lead to apoptosis in affected tissue and primary culture of human cells that harbor mtDNA mutations [66]. There are several studies which point to mitochondrial dysfunction in glaucoma and RGC death [66-68]. One hypothesis suggests that progressive optic nerve damage in POAG is the result of optic nerve fiber apoptosis [67]. Mitochondria-induced apoptosis, which may be a mechanism of injury in experimental glaucoma [67] and other optic neuropathies [66], may also be a pathological factor in PCG. Recent study by Abu-Amero et al. [11] reported mitochondrial dysfunction-associated OS as a risk factor for glaucoma. MtDNA alterations result in reduced mitochondrial respiration [11] and OS [36]. Thus reduced ATP levels secondary to mitochondrial damage may impair development and differentiation of TM. Endothelial cells are also damaged due to supraphysiological ROS levels.

These findings suggest that elevation of IOP is related to oxidative degenerative processes affecting the TM specifically endothelial cells. Much evidence indicates that in this region ROS play a fundamental pathogenic role by reducing local antioxidant activities inducing outflow resistance. TM is neural crest in origin [69,70] and developing TM is deficient in antioxidant enzymes and more susceptible to OS induced DNA damage [71]. OS disturbs Ca^2+^ homeostasis and so raised Ca^2+^ levels activate endonucleases which cause nuclear DNA damage [63]. OS, early in development and/or throughout life could precipitate both metabolic and anatomic sequelae that cause trabecular dysgenesis and ultimately optic nerve damage in PCG.

**Figure 4:**
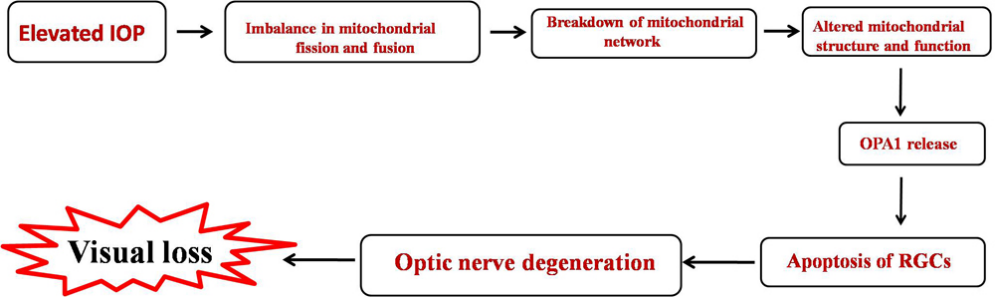
Role of elevated IOP in RGCs death in glaucoma. Abbrevations: RGCs- Retinal ganglion cells; IOP- intra ocular pressure; OPA1-optic nerve atrophy type-I.

Elevated IOP is a characteristic feature of glaucoma and an important risk factor for optic nerve damage [72]. However, the precise relationship between among elevated IOP, glaucomatous optic nerve (ON) damage, and retinal ganglion cell death are poorly understood. Growing evidence indicates that mitochondrial structural and functional dynamics play an important role in cell and animal physiology. Imbalance in the control of mitochondrial fusion and fission dramatically alters overall mitochondrial morphology [73-76]. Elevated IOP in glaucoma induces reduction of cytochrome c oxidase (COX) activity, mitochondrial fission, mitochondrial matrix swelling, cristae depletion, triggers release of optic nerve atrophy type-I (OPA1), and induces subsequent apoptotic cell death in differentiated RGC-5 cells [77,78] (Figure 4). Similar findings were also confirmed in a mouse model [79].

In summary, frequency of mtDNA sequence variations in PCG was significantly higher as compared to controls. Five pathogenic changes (3 in *ND1* and 2 in *ND2*) and 3 other changes (G10398A, A12308G, and G13708A) associated with elevated ROS were present in our patients. Non-synonymous mtDNA alterations may lead to mitochondrial dysfunction which leads to reduced mitochondrial respiration, OS, damage to mtDNA, altered mitochondrial morphology, alterations in mitochondrial fission and fusion, and ultimately cell’s demise. OS impairs development and differentiation of trabecular meshwork that favor increased intraocular pressure in PCG and consequently RGC death.

This study describes mtDNA sequence variations in a relatively small number of patients with PCG of north Indian ethnic origin. However, these results should be confirmed in other populations. Knowledge of mtDNA mutations and/or mitochondrial dysfunction in PCG may lead to a better understanding of glaucoma pathophysiology [80]. Novel approaches are now available for studying mitochondrial disease in the eye, and a novel in vitro treatment has already been devised for the metabolic defect of at least one mtDNA mutation in LHON [81]. PCG cases with mtDNA variations and consequent OS may benefit by early diagnosis and prompt management with antioxidant therapy.

### Conclusion

A total of 44 novel mtDNA variations were identified in current study. MtDNA variations adversely affect respiratory chain, impair the OXPHOS pathway resulting in low ATP production, and impair growth, development, and differentiation of TM. Mitochondrial DNA variations also lead to increased ROS production, oxidative injury to TM and RGCs. Thus, early diagnosis of mitochondrial DNA variations and prompt antioxidant administration may delay OS induced injury to TM and RGCs and hence improve visual prognosis.
